# Generic drug crisis in Japan and changes leading to the collapse of universal health insurance established in 1961: the case of Kobayashi Kako Co. Ltd.

**DOI:** 10.1186/s12962-023-00441-z

**Published:** 2023-05-31

**Authors:** Makoto Kosaka, Akihiko Ozaki, Yudai Kaneda, Hiroaki Saito, Erika Yamashita, Anju Murayama, Hanano Mamada, Tetsuya Tanimoto, Mihajlo Jakovljevic

**Affiliations:** 1 Imamura Hospital Kagoshima, Kagoshima, Japan; 2 Medical Governance Research Institute, Minato-ku, Tokyo, Japan; 3 Department of Breast and Thryoid Surgery, Jyoban Hospital of Tokiwa Foundation, Iwaki, Fukushima Japan; 4 School of Medicine, Hokkaido University, Sapporo, Hokkaido Japan; 5 School of Medicine, Tohoku University, Sendai, Miyagi Japan; 6 Institute of Advanced Manufacturing Technologies, Peter the Great St. Petersburg Polytechnic University, St. Petersburg, Russia; 7 Institute of Comparative Economic Studies, Hosei University, Chiyoda-ku, Tokyo, Japan; 8 Department of Global Health Economics and Policy, University of Kragujevac, Kragujevac, Serbia

Japan established a national health insurance scheme in 1961, and thanks to this, all the Japanese population have equal access to decent medical care by paying only 10–30% of the medical cost from their own pocket basically, depending on the person's age [1]. An overlooked consequence was ever-increasing healthcare costs. With the advancement of medical technology and a fast-aging society, Japan’s healthcare cost has kept rising, exceeding 44 trillion JPY in 2019, which calls for an overhaul of its health insurance scheme [2]. However, the Japanese government failed to offer a fundamental solution and instead took a temporary measure in 2015 to encourage the use of generic drugs.

Consequently, the percentage of generic drugs in total amount prescriptions increased from 32.5% in 2005 to 78.3% as of September 2020 [3]. However, we cannot help saying that a consequence of this policy change has been problematic, as the country has recently faced an unprecedented series of incidents related to poor quality assurance. The representative case is that, in 2020, an oral antifungal drug itraconazole, sold by a Japanese generic drug company Kobayashi Kako Co., Ltd, was found to be accidentally contaminated with a sleep-inducing ingredient during their manufacturing process. At least 245 patients taking the drug had unwarranted side effects, including two deaths due to loss of consciousness while driving.

An investigation revealed that the company had manufactured pharmaceuticals through unauthorized procedures since 2005. It was divulged that, since the late 1970s, the company had not conducted any quality tests and had fabricated the results. Additionally, only 800 staff were allocated to 500 types of products, indicating significant understaffing to underpin the various production. As a result of this incident, on 9 February 2021, the company was ordered by Fukui Prefectural office to suspend operations for 116 days, the longest suspension ever for a pharmaceutical company.

Additionally, the company was also found to have made false statements in its applications for pharmaceutical approvals for 12 products, and in April 2021, the Ministry of Health, Labour, and Welfare of Japan revoked their approvals and ordered it to improve its operations [4]. A series of these scandals have critically damaged the company's financial status and reputation and led to its bankruptcy. As a result, the company was taken over by the Japanese second largest generic drug company Sawai Pharmaceutical Co., Ltd, in March 2022, with 2020 sales of 182,537 million JPY [5].

Not only the case above, the largest generic drug manufacturer in Japan Nichi-Iko Pharmaceutical Co., Ltd., with its 2020 sales of 190,076 million JPY, was found to have been selling its products with unauthorized procedures since 2009 and was ordered to suspend operations in March 2021. After this incident, inspections by prefectures were strengthened, and a certain number of pharmaceutical companies were ordered to suspend production for similar violations. Sudden suspensions of production because of such problems threaten stable supply, and there were repetitive shortages and replacements of various types of generic drugs including essential medicines, causing confusion in daily clinical practice [6]. As of August 2021, out of 15,433 medications, 743 had their shipments suspended and 2400 had been undershipped [7]. Shortages of essential medicines in other wealthy OECD markets have been documented to lead to substantial opportunistic costs of medical care [8]. Various strategies to tackle these bottleneck weaknesses have been proposed [9]. Yet one should keep in mind that Japanese advanced pharmaceutical technology innovation [10] and diversity of dosage forms of approved medicines exceeding the Western one by a strong degree [11]. These facts add certain complexity to the challenge [12].

One of the reasons behind these incidents is the Japanese government's policies to reduce medical costs in order to protect the country's universal health coverage, which is in a state of crisis due to the super aging society [13]. The government has started giving manufacturers and distributors permission independently since 2005 with the revision of the Pharmaceutical Affairs Law, which prompted pharmaceutical companies to outsource their drug manufacturing function. Consequently, barriers to entry into contract production of generic drugs were lowered, and chemical manufacturers or textile manufacturers, etc. started production of generic drugs in Japan [14]. As a result, the total number of generic drug companies has increased to 194 as of November 2019, with the majority being small-scale companies.

However, their sustainability has increasingly become questionable as the frequency of the official drug price revision once ever year since 2021 has made it difficult for generic companies to restructure their portfolio and gain sufficient profit constantly, jeopardizing thier financial status. Indeed, the promotion policies advocating the usage of generic drugs have prompted companies to increase their investments in generic drug manufacturing, thereby assisting Japan in approaching its generic target of 80% (78.3% as of September 2020). However, in reality, not only has the expansion of factories and personnel failed to keep pace, but their financial status has also become increasingly fragile and vulnerable to policy changes concerning generic drugs. In fact, as for the medical cost, the government estimates that generic drugs reduced the expenditure by about 17 billion USD in 2020, though the recent annual drug expenditure has been around 92 billion USD in Japan. On the other hand, in this situation, generic manufacturers have been forced to expand production to meet the demand while prioritizing stable supply with thin profit, neglecting sufficient quality assurance. There is an obvious trade-off between the quality against the cost of manufacturing and regulatory requirements of these products [15].

Although the government has conducted an unannounced on-site inspection to ensure quality assurance and improve domestic regulations to match Good Manufacturing Practice global standards, it would not be easy to regain trust in generic drugs. The reckless promotion policy of generic drugs should be reconsidered so that generic drug companies can achieve economical sustainability [16], and additional costs should be accepted to produce reliable generic drugs. These recommendations would essentially go into the footsteps of decades-long Japanese policy to protect brand name medicines [17]. Such policy rewards the cost of innovation via 5 years of extensions of post-patent exclusivity after license expiration [18]. Thus, we firmly believe an effective pharmaceutical policy equilibrium must satisfy both Japanese public sentiment for quality and copy-cat pharmaceutical manufacturers’ needs for survival in a highly competitive market [19].

## Data Availability

All data cited in this contribution are publicly accessible.
